# Real-Time Optical Diagnosis of the Rat Brain Exposed to a Laser-Induced Shock Wave: Observation of Spreading Depolarization, Vasoconstriction and Hypoxemia-Oligemia

**DOI:** 10.1371/journal.pone.0082891

**Published:** 2014-01-08

**Authors:** Shunichi Sato, Satoko Kawauchi, Wataru Okuda, Izumi Nishidate, Hiroshi Nawashiro, Gentaro Tsumatori

**Affiliations:** 1 Division of Biomedical Information Sciences, National Defense Medical College Research Institute, Tokorozawa, Saitama, Japan; 2 Graduate School of Bio-Application and Systems Engineering, Tokyo University of Agriculture and Technology, Koganei, Tokyo, Japan; 3 Division of Neurosurgery, Tokorozawa Central Hospital, Tokorozawa, Saitama, Japan; 4 Department of Defense Medicine, National Defense Medical College, Tokorozawa, Saitama, Japan; University of South Florida, United States of America

## Abstract

Despite many efforts, the pathophysiology and mechanism of blast-induced traumatic brain injury (bTBI) have not yet been elucidated, partially due to the difficulty of real-time diagnosis and extremely complex factors determining the outcome. In this study, we topically applied a laser-induced shock wave (LISW) to the rat brain through the skull, for which real-time measurements of optical diffuse reflectance and electroencephalogram (EEG) were performed. Even under conditions showing no clear changes in systemic physiological parameters, the brain showed a drastic light scattering change accompanied by EEG suppression, which indicated the occurrence of spreading depression, long-lasting hypoxemia and signal change indicating mitochondrial energy impairment. Under the standard LISW conditions examined, hemorrhage and contusion were not apparent in the cortex. To investigate events associated with spreading depression, measurement of direct current (DC) potential, light scattering imaging and stereomicroscopic observation of blood vessels were also conducted for the brain. After LISW application, we observed a distinct negative shift in the DC potential, which temporally coincided with the transit of a light scattering wave, showing the occurrence of spreading depolarization and concomitant change in light scattering. Blood vessels in the brain surface initially showed vasodilatation for 3–4 min, which was followed by long-lasting vasoconstriction, corresponding to hypoxemia. Computer simulation based on the inverse Monte Carlo method showed that hemoglobin oxygen saturation declined to as low as ∼35% in the long-term hypoxemic phase. Overall, we found that topical application of a shock wave to the brain caused spreading depolarization/depression and prolonged severe hypoxemia-oligemia, which might lead to pathological conditions in the brain. Although further study is needed, our findings suggest that spreading depolarization/depression is one of the key events determining the outcome in bTBI. Furthermore, a rat exposed to an LISW(s) can be a reliable laboratory animal model for blast injury research.

## Introduction

There are many risks for humans to encounter blast or shock waves in military, industrial and even natural environments. Most seriously, terrorism using explosive devices has been increasing in recent years, resulting in a large number of patients suffering from blast-induced traumatic brain injury (bTBI) [1]–[3]. Many of them show no evident abnormality by conventional imaging diagnoses, such as MRI and X-ray CT, but are troubled with higher order brain dysfunction as well as post-traumatic stress disorder (PTSD) in the chronic phase, which is called mild bTBI (mbTBI) [4]–[7]. However, the pathophysiology and mechanism of blast-related TBI are not well understood. Natural blast or shock waves are caused by volcanic eruptions, lightning and meteorites. The recent explosion of a meteorite in Chelyabinsk, Russia resulted in injuries to more than one thousand people [8], and the outcomes of their injuries are unpredictable. Thus, comprehensive investigation is needed to establish methods for prevention, diagnosis and treatment of bTBI, for which experiments using animal models are needed.

It is known that complex effects are involved in bTBI. These effects include the effect of the blast wave itself (primary mechanism), the effect of missiles being propelled by the blast force (secondary mechanism), and the effect of impacts with other objects (tertiary mechanism) [9]. The secondary and tertiary mechanisms are relatively well understood and documented. On the other hand, the primary mechanism, the effect of the blast wave itself, has not been elucidated and would be closely associated with higher order brain dysfunction due to bTBI. In fluid dynamics, a blast wave means the pressure and flow resulting from an explosion-like event, which has a leading shock wave (shock front) followed by a wind of negative pressure [10]. All of the components of a blast wave can affect living tissue, but its shock wave component, which is characterized by an extremely fast pressure rise and high peak pressure, is considered to be most invasive. Thus, it is essential to use a real shock wave to investigate bTBI with animals. Standard TBI animal models, such as those produced by weight drop [11]–[16], fluid percussion [17]–[19] and controlled cortical impact [20], [21], are useful to study contusion-type TBI, but their pressure characteristics are totally different from those of real shock waves.

As real shock wave sources, micro actual explosions [22], shock [23]–[27]/blast [28] tubes, and air gun [29]–[31] have so far been used to study bTBI in laboratories. We have been proposing the use of laser-induced shock waves (LISWs) to investigate blast injuries. Satoh *et al.* first used LISWs to mimic blast-induced lung injury in mice [32]. Hatano *et al.* applied an LISW to the rat brain through a cranial window and analyzed the brain tissue histologically [33]. Advantages in using LISWs for bTBI study include safety, ease to use, compact device, versatility and highly controllable shock wave energy, both temporally and spatially. These characteristics enable many unique animal experiments that cannot be performed with other shock wave sources.

In this study, we performed real-time optical diagnosis of the rat brain that was exposed to an LISW. Simultaneously, electroencephalogram (EEG) was recorded and systemic physiological parameters were measured. Under the standard LISW conditions examined in this study, no clear changes were observed in systemic physiological parameters, and hemorrhage and contusion were not apparent in the cortex. However, the brain showed a drastic change in the light scattering signal, EEG suppression, long-lasting hypoxemia and signal change indicating mitochondrial energy impairment. The light scattering change accompanied by EEG suppression indicated the occurrence of spreading depression, and we examined related events to confirm this. We observed a distinct negative DC potential shift (spreading depolarization), time-dependent expansion of the hypoxemic region, and vasodilatation followed by long-lasting vasoconstriction. Computer simulation showed that hemoglobin oxygen saturation reached as low as ∼35% in the hypoxemic phase. In this paper, we describe the results of these multimodal real-time measurements for the rat whose brain was exposed to an LISW and discuss their cross-correlation.

## Materials and Methods

The Ethics Committee of Animal Care and Experimentation, National Defense Medical College, Japan, approved all requests for animals and the intended procedures of the present study (Permission number: 10040). All animal experiments were performed under anesthesia, as described later, and all efforts were made to minimize suffering. After experiments, animals were sacrificed with an overdose of pentobarbital sodium (150 mg/kg i.p.).

### Generation and characteristics of LISW

**Figure 1:**
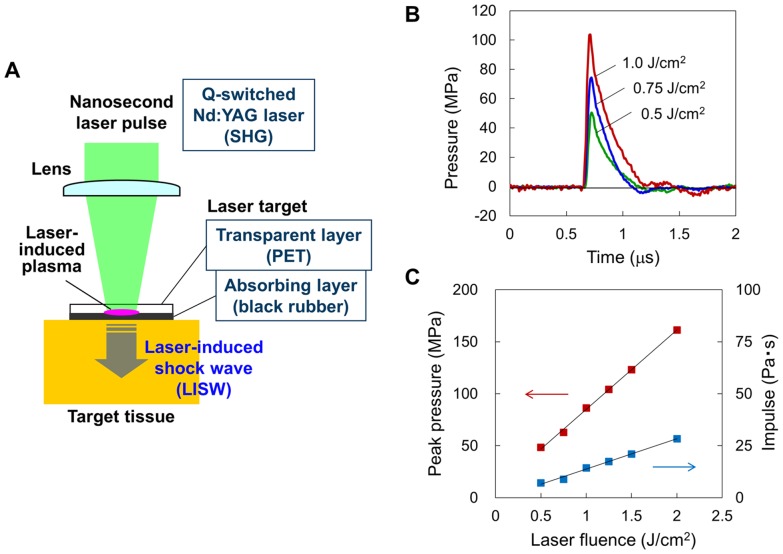
Generation and characteristics of laser-induced shock wave (LISW). (A) Setup for generating an LISW. (B) Typical temporal waveforms of LISWs generated at different laser fluences on the laser target. (C) Dependences of peak pressure and impulse of LISW on laser fluence.

Figure 1A shows the method for generation of an LISW. A laser target, which is a laser-absorbing material (0.5-mm-thick natural black rubber disk) to which an optically transparent material (1.0-mm-thick transparent polyethyleneterephthalate sheet) is adhered, is placed on the tissue. The target is irradiated with a short laser pulse, which is absorbed by the rubber to induce a plasma, and its expansion is accompanied by a shock wave (LISW). The polyethyleneterephthalate (PET) sheet has a role to confine the plasma, by which peak pressure and impulse of the LISW can be increased. As a laser source, nanosecond pulsed laser is suitable, since its laser pulse temporally overlaps well with its own inducing plasma, by which the plasma can absorb the laser energy, causing an increase in plasma energy and hence pressure of the LISW. In this study, the second harmonics of a Q-switched Nd:YAG laser (Brilliant b, Quantel, Les Ulis Cedex, France; wavelength, 532 nm; pulse width, 6 ns) was used. The scheme used in this study was the same as those used for drug and gene delivery studies, where the pressure wave was synonymically called photomechanical wave (PMW) or laser-induced stress wave [34]–[39]. In those studies, excessive increase in the laser fluence and hence pressure caused tissue damage, which gave us an idea of using LISWs to investigate blast injuries.

Figure 1B shows typical temporal pressure profiles of LISWs generated at different laser fluences on the target, which were measured with a needle type hydrophone (HNR-1000, Onda Co., Sunnyvale, CA) that was placed under the target. The waveforms are characterized by fast rise time, high peak pressure, short duration (a few hundreds of nanoseconds in FWHM) and positive pressure dominance. As described above, LISWs have high controllability of shock wave energy; the peak pressure and impulse linearly increase with increasing laser fluence (Fig. 1C) and the size of the wave source can easily be changed by changing the laser spot size on the target.

A common question about the use of LISWs to investigate blast injuries is the similarity with actual explosions. Peak pressures and durations of medically relevant actual explosions would be in the ranges of 100 kPa–1 MPa and 0.1 ms–10 ms, respectively [40]; the peak pressures are roughly three orders of magnitude higher, while the durations are three orders of magnitude shorter than those of LISWs. Thus, impulses (time-integrated positive pressures) of medically relevant actual explosions and LISWs used in this study are in the same order. Since the impulse is one of the primary factors to determine the degree of pressure-induced tissue damage [41], [42], we think that LISWs can be used to simulate TBIs induced by an actual explosion. If needed, however, the temporal characteristics of an LISW (pressure waveforms and duration) can be modified by the insertion of various materials between the laser target bottom surface and tissue surface.

### Animal preparation and setup for LISW application, optical diagnosis and EEG measurement for the brain

A Sprague-Dawley male rat (Japan SLC, Hamamatsu, Japan) weighing 310 to 380 g was anesthetized with pentobarbital sodium (50 mg/kg animal weight) and placed in a stereotactic frame (Narishige, Tokyo, Japan). An LISW can be applied to the brain either through the scalp, transcranially or directly through a cranial window. In this study, transcranial application was used. After the head had been shaved, the scalp was incised at the midline and the parietal bone was exposed.

**Figure 2:**
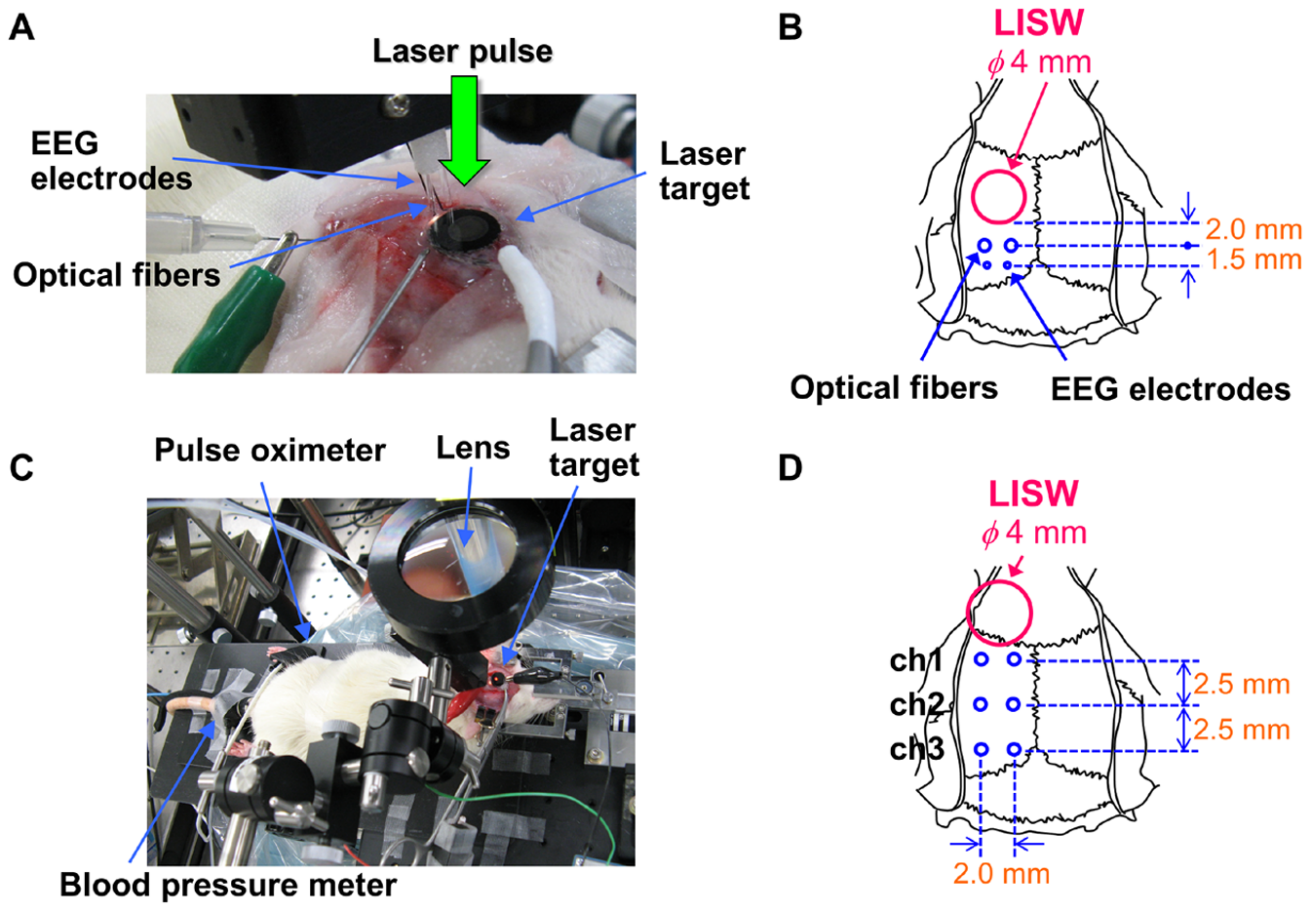
Measurements of diffuse reflectance signals, EEG and systemic physiological parameters for the rat whose brain was exposed to an LISW. (A) Photograph of setup and (B) schematic of sensor positions for measurements of diffuse reflectance signals and EEG for the brain. (C) Photograph showing sensor positions for pulse oximeter and blood pressure meter. (D) Positions of fiber pairs (ch1, ch2 and ch3) and LISW application for multichannel diffuse reflectance measurement of the brain.

Figure 2A and 2B shows a photograph and schematic arrangement of the setup for the application of an LISW, optical diagnosis and EEG measurement for the brain. A laser target (rubber disk of 8 mm in diameter covered with a PET sheet) was held with forceps and placed in the anterior region of the parietal bone; ultrasound gel was used between the bottom of the rubber and skull for acoustic impedance matching. In most experiments described in this paper, laser spot size on the target and hence diameter of the LISW was kept constant at 4 mm. In the vicinity of the laser target, a pair of optical fibers (core diameter, 800 µm; center to center distance of fibers, 2 mm) was placed on the skull for optical diagnosis, and electrodes for EEG measurement (250-µm-diameter stainless steel needle type) were inserted in the skull. The distances from the edge of the LISW to the fibers and to the electrodes were approximately 2 mm and 3.5 mm, respectively. The optical diagnosis is described in detail in the next section.

### Real-time optical diagnosis of the rat brain

We performed fiber-based diffuse reflectance measurement for the brain; the method was similar to the methods used in our previous studies [43], [44]. Broadband (400–1000 nm) light from a tungsten lamp (BPS120, B&W Tek Inc., Newark, DE) was delivered to the brain through one of the fibers described above. The light penetrated through the skull and reached the cortex. A part of the diffused light in the cortex was captured with another fiber and it was transmitted to a polychromator (PMA-11, Hamamatsu Photonics, Hamamatsu, Japan). The diffused light includes information on scattering and absorption properties of the tissue; light scattering mainly reflects morphological characteristics of cells and organelles [45], while light is absorbed by chromophores in the tissue, such as hemoglobins and redox centers in cytochrome c oxidase (CcO) of the mitochondrial electron transport chain. Thus, information on morphological characteristics, hemodynamics and mitochondrial energy metabolism in the cortex can be obtained by spectroscopic analysis of the diffused light. Our Monte Carlo simulation showed that the cortex in the depth range of 0.8–1.5 mm can be analyzed by the present fiber configuration [43].

In this study, we observed light scattering signal at the wavelength of 805 nm (R_805_) in the near-infrared (NIR) region, where the reduced scattering coefficient of the gray matter is two orders of magnitude greater than the absorption coefficient [46] and, thus, morphological characteristics of the tissue can be measured with high contrast. As a signal of total hemoglobin concentration, we used the reflectance signal at 569 nm (R_569_), which is an isosbestic point of oxyhemoglobin and deoxyhemoglobin. This signal indicates regional cerebral blood volume (rCBV) with the assumption of a constant hematocrit value. To know the hemoglobin oxygenation level, the reflectance signal at 578 nm, where the absorption of oxyhemoglobin peaks, was normalized by the reflectance signal at 569 nm (R_578_/R_569_). To obtain information on the mitochondrial energy metabolism, optical absorption characteristics of heme aa_3_, a redox center of CcO, was used. There is an isosbestic point for reduced and oxidized heme aa_3_ at 620 nm, while absorption of reduced heme aa_3_ is maximized at 605 nm [47]. Thus, the reflectance signal at 605 nm that is normalized by the reflectance signal at 620 nm (R_605_/R_620_) can indicate the state of mitochondrial energy metabolism. The light scattering signal (R_805_) and signal indicating rCBV (R_569_) were normalized by the values before LISW application. All of these reflectance signals, as well as EEG signal, were simultaneously measured before, during and after application of an LISW to the brain.

### Measurement of systemic physiological parameters

Simultaneously with the above-described optical and EEG measurements for the brain, systemic physiological parameters were measured for the rat (Fig. 2C). Arterial oxygen saturation (SpO_2_) was monitored with a pulse oximeter (8600 V, Nonin Medical, Inc., Plymouth, MN), for which the probe was attached to the forelimb. Heart rate and mean arterial pressure were measured with a noninvasive blood pressure monitor system (MK-2000ST, Muromachi, Tokyo, Japan), for which the sensor was placed at the root of the tail.

### Observation of spreading depression-related events

As is described in Results, we observed a drastic change in the light scattering signal (R_805_) just after LISW application by fiber-based measurement. The scattering signal change was accompanied by EEG suppression, indicating the occurrence of spreading depression. Spreading depression means silencing of brain electrical activity as a consequence of spreading depolarization. Spreading depolarization is characterized by abrupt, near-complete sustained depolarization of neurons, which can be observed as a large change of DC potential or slow potential. It is known that spreading depression propagates over the cortex together with spreading depolarization [48].

To confirm the occurrence of spreading depolarization and its correlation with light scattering change, we measured DC potential and light scattering intensity. For DC potential measurement, a small hole (diameter of ∼2 mm) was made at about the same position as that for the EEG electrodes (Fig. 2B) and an electrode (1-mm-diameter Ag-AgCl ball type) was placed on the brain surface. For measurement of light scattering intensity, the brain was illuminated through the skull with NIR light emitted from a white light lamp (HL 100E, HOYA- Schott, Tokyo, Japan) with a bandpass filter (800±70 nm). Immediately after laser irradiation to generate an LISW (t = 0 s), the laser target was quickly removed and the diffusely reflected NIR light from the brain was imaged with an 8-bit CCD (XC-HR57, Sony, Tokyo, Japan). On the basis of the CCD image, light intensity in the region of interest (ROI) adjacent to the site of the electrode for DC potential measurement was integrated and its time course was compared with that of the DC potential. We also visualized propagation of a scattering wave, for which difference images, the image immediately after LISW application being subtracted from the image at time t, were made to enhance the contrast, creating a video movie of the scattering wave.

Furthermore, we observed vasculature response to LISW in the brain. The head of the rat was fixed with a stereotactic frame (Narishige, Tokyo, Japan) and a cranial window of 4 mm in diameter was made. The window was fitted with a 0.5-mm-thick optically transparent plastic (PET) disk of the same diameter, and its edge was sealed with dental cement (RelyX Unicem, 3M Deutschland, Neuss, Germany). The rat brain was placed under a stereoscopic microscope (SteREO Lumar.V12, Carl Zeiss, Jena, Germany) and blood vessels in the brain surface were observed with a high-resolution color CCD (AxioCam HRc, Carl Zeiss, Jena, Germany) before and after LISW application.

### Spatiotemporal correlation between spreading depression and hypoxemia

We expected that spreading depolarization/depression (SD) caused hypoxemia in the brain and assumed that the region of hypoxemia expanded along with the propagation of SD. To examine this speculation, we assessed the spatiotemporal correlation between SD and hypoxemia for the brain, for which multichannel fiber-based diffuse reflectance measurement was performed. The laser target was placed on the frontal bone and three pairs of fibers, which were placed at positions with the different distances, 2.0 mm (ch1), 4.5 mm (ch2) and 7.0 mm (ch3), from the edge of the LISW source were used (Fig. 2D). Light from the same tungsten lamp was equally divided and coupled to three illumination fibers. Reflectance signals transmitted through the detection fibers were delivered to the polychromator through a mechanical optical switch (Fiber optical multimode switch MOL 1×4, LEONI Fiber Optics, Neuhaus-Schierschmitz, Germany). Time course of the value R_578_/R_569_ indicating hemoglobin oxygenation level was measured at the three positions, by which expansion speed for the region of hypoxemia was estimated.

### Quantification of hemoglobin oxygen saturation

As described above, we monitored hemoglobin oxygenation level by fiber-based measurement, but it only gave information on relative change. Thus, on the basis of the measured diffuse reflectance data, we quantified hemoglobin oxygen saturation (*StO*_2_) by inverse Monte Carlo simulation (MCS). The method is described in detail in Ref. 44, but briefly, we used multiple regression analysis aided by MCS for the diffuse reflectance spectra. The measured reflectance spectrum *R*(*λ*) was first converted to an absorbance spectrum *A*(*λ*), which was defined as *A*(*λ*) = −log_10_ *R*(*λ*). By using the absorbance spectrum as a response variable and extinction coefficients of oxygenated hemoglobin *ε*_HbO_(*λ*) and deoxygenated hemoglobin *ε*_HbR_(*λ*) as predictor variables, we performed multiple regression analysis, providing regression coefficients: α_HbO_, α_HbR_ and α_0_. The coefficients α_HbO_ and α_HbR_ represent the degree of contribution of each extinction coefficient to the absorbance spectrum and the coefficient α_0_ is related to the degree of contribution of the attenuation due to light scattering in the brain to the absorbance spectrum. From these regression coefficients, the concentrations of oxygenated hemoglobin *C*_HbO_ and deoxygenated hemoglobin *C*_HbR_ and scattering amplitude *a* were determined by using conversion vectors that had been prepared numerically in advance. The scattering amplitude *a* is a proportional constant of reduced scattering coefficient *μ*_s_′(*λ*) of brain tissue. By using these hemoglobin concentrations *C*_HbO_ and *C*_HbR_, we obtained the value of hemoglobin oxygen saturation (*StO*_2_) based on the following equation: *StO*_2_ = *C*_HbO_/(*C*_HbO_+*C*_HbR_).

In this simulation, we found that optical properties of the skull greatly affected the results, while their accurate measurement is difficult *in vivo*. Thus, we made a separate optical measurement for the rat brain, for which we made a 1 mm×3 mm rectangular window in the skull and placed a pair of optical fibers directly on the cortex. A laser target was then placed in the vicinity of the window and was irradiated with a laser pulse to generate an LISW; the relative positions of the fiber and target were the same as those shown in Fig. 2B.

## Results

### Systemic physiology, EEG and diffuse reflectance signals for the brain

**Figure 3:**
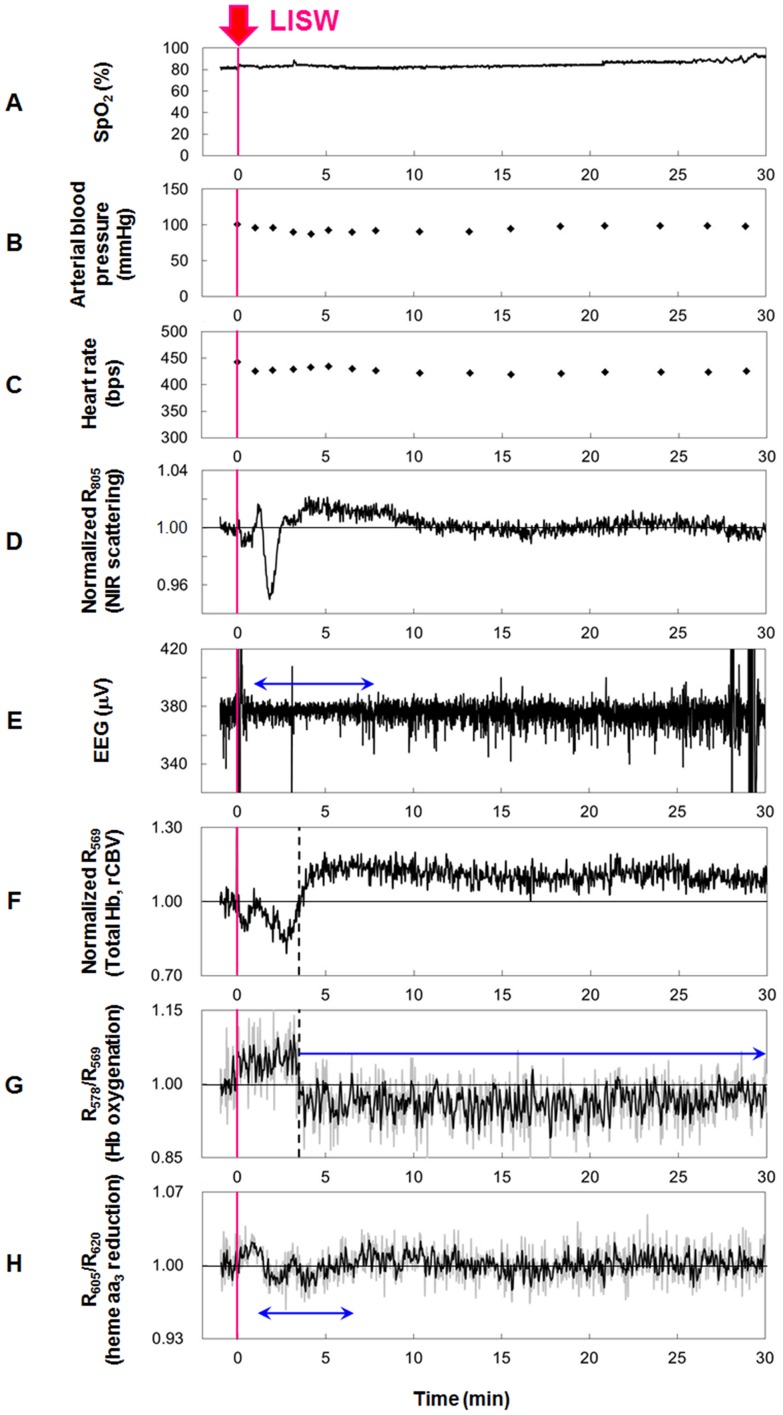
Results of measurements of systemic physiological parameters, EEG and diffuse reflectance signals for the brain. Sensor positions for systemic physiological parameters (A–C), EEG (E) and diffuse reflectance signals (D, F–H) are shown in Fig. 2. A single pulse of LISW generated at 1.0 J/cm^2^ (*φ*4 mm; ∼86 MPa; ∼14 Pa•s) was applied to the brain at time zero. (A) Arterial oxygen saturation (SpO_2_). (B) Arterial blood pressure. (C) Heart rate. (D) Light scattering signal (diffuse reflectance signal at 805 nm, R_805_) indicating cellular and subcellular morphological changes. (E) EEG. The horizontal arrow indicates the duration of EEG suppression. (F) Total hemoglobin indicating regional cerebral blood volume (rCBV) (R_569_). The vertical dashed line indicates the turning point from hyperemia to oligemia. (G) Hemoglobin oxygenation (R_578_/R_569_). The vertical dashed line indicates the turning point from hyperoxemia to hypoxemia. The horizontal arrow indicates long-lasting hypoxemia. (H) Diffuse reflectance signal indicating reduction of heme aa_3_, a redox center of cytochrome c oxidase (R_620_/R_605_). The horizontal arrow indicates the duration of heme aa_3_ reduction.

Figure 3 shows the results of measurements of systemic physiological parameters, EEG and diffuse reflectance signals for the rat whose brain was exposed to an LISW generated at a laser fluence of 1.0 J/cm^2^ (average peak pressure, ∼86 MPa; average impulse, ∼14 Pa•s) at time zero (t = 0). No remarkable change was observed in SpO_2_, blood pressure or heart rate (Fig. 3A–C), while drastic changes were observed in the signals measured for the brain. Scattering signal (R_805_) changed vigorously for about 4 min after LISW application (Fig. 3D), indicating drastic cellular and subcellular morphological changes at the site of measurement in the cortex. This was accompanied by suppression of the EEG for about 7 min (Fig. 3E). These observations indicate the occurrence of spreading depression. Its confirmation and results of the observation of related events are described later.

During a period of about 4 min after LISW application, the rCBV signal (R_569_) decreased (Fig. 3F), indicating an increase in total hemoglobin concentration and hence hyperemia, and the hemoglobin oxygenation signal (R_578_/R_569_) increased (Fig. 3G), indicating hyperoxemia. Thereafter, however, both signals began to change oppositely, showing a long-lasting (∼1.5 h) oligemia (Fig. 3F) and hypoxemia (Fig. 3G). In the same measurements for other rats, decreased rCBV and hypoxemia lasted for more than 1 h in many cases and for 3 h in the longest case. We also measured oxygen partial pressure with a Clark-type polarographic electrode (PO2eD-405DN, Bio Research Center, Nagoya, Japan) in the brain. Oxygen partial pressure decreased by ∼40% in the hypoxemic phase, indicating the occurrence of hypoxemic hypoxia in the brain (data not shown). Long-term hypoxemia or ischemia is known to be an important symptom in patients with bTBI [49]. The results of quantification of hemoglobin oxygen saturation are described later. About 1.5 min after LISW application, we also observed a decrease in the heme aa_3_-related signal, R_605_/R_620_, for ∼3 min (Fig. 3H), indicating reduction of heme aa_3_, a sign of mitochondrial energy impairment.

We performed the same measurements in the laser fluence range of 0.75–1.5 J/cm^2^, the corresponding average peak pressure and impulse ranges being ∼63–∼123 MPa and ∼9–∼23 Pa•s, respectively. After the measurements, brains were exposed or extracted. In some cases, hemorrhage was observed in the dura mater, but there was no or little hemorrhage in the cortex. Histological examination with hematoxylin and eosin staining showed no remarkable contusion or laceration in the brain under any of the LISW conditions (data not shown). Under the standard LISW conditions (fluence, 1.0 and 1.25 J/cm^2^), disruption of the blood brain barrier (BBB) was examined by intravenous injection of Evans Blue (EB), and intracranial pressure (ICP) was measured with a fiber optic pressure sensor (FISO-LS-PT9, FISO Technologies, Quebec, Canada). In the acute phase (within 1 h after LISW application), extravasation of EB was not visible and change in ICP was not evident for the brains.

Table 1 summarizes main observations showing whether drastic scattering change, EEG suppression, long-lasting hypoxemia and signal change indicating heme aa_3_ reduction were observed or not. At a laser fluence of 1.0 J/cm^2^ (average peak pressure, ∼86 MPa; average impulse, ∼14 Pa•s), we observed drastic scattering change accompanying EEG suppression for 9 of 12 rats. For 8 of those 9 rats, prolonged hypoxemia was observed, and heme aa_3_ reduction was indicated in 6 of those 8 rats. Probabilities of the occurrence of these events seem to increase with increasing peak pressure and impulse. In the total of 24 rats examined, drastic scattering change accompanying EEG suppression, which indicated the occurrence of spreading depression, was observed for 18 rats; prolonged hypoxemia was observed in 15 (83.3%) of those 18 rats and heme aa_3_ reduction was indicated in 14 (77.8%) of those 18 rats, showing a strong correlation between these events.

**Table 1:** Summary of observations by measurements of diffuse reflectance signal and EEG; mark (X) indicates observed.

Fluence (J/cm^2^)	Average peak pressure (MPa)	Average impulse (Pa•s)	Rat#	Drastic light scattering change	EEG suppression	Long-lasting hypoxemia	Signal change indicating heme aa_3_ reduction
0.75	∼63	∼9	1				
			2	X	X	X	X
			3				
			4	X	X	X	X
			5				
1.0	∼86	∼14	1	X	X		
			2				
			3	X	X	X	X
			4	X	X	X	
			5	X	X	X	X
			6	X	X	X	X
			7	X	X	X	
			8	X	X	X	X
			9				
			10				
			11	X	X	X	X
			12	X	X	X	X
1.25	∼104	∼19	1	X	X	X	X
			2	X	X	X	X
			3	X	X	X	X
			4	X	X		X
			5	X	X	X	X
1.5	∼123	∼23	1	X	X		
			2	X	X	X	X

### DC potential shift and light scattering change

**Figure 4:**
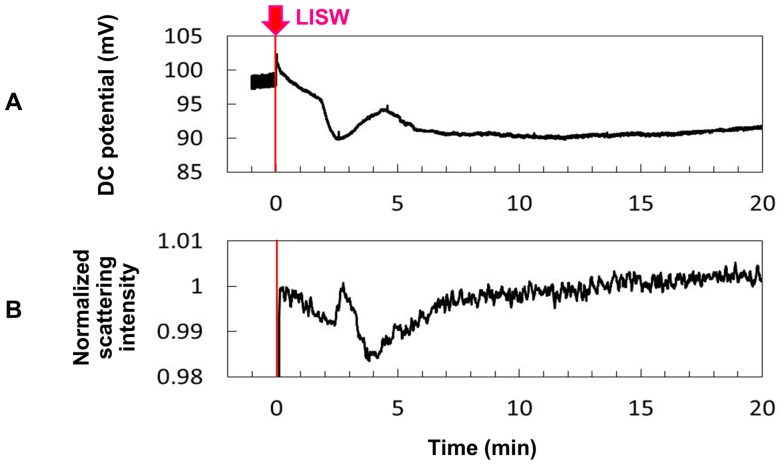
Results of measurements of DC potential and light scattering signal based on CCD imaging. A single pulse of LISW generated at 1.0/cm^2^ (*φ*4 mm; ∼86 MPa; ∼14 Pa•s) was applied to the brain at time zero. (A) DC potential measured at the same position for EEG measurement (Fig. 2B). (B) Light scattering intensity in the ROI adjacent to the site of DC potential measurement.

**Figure 5:**
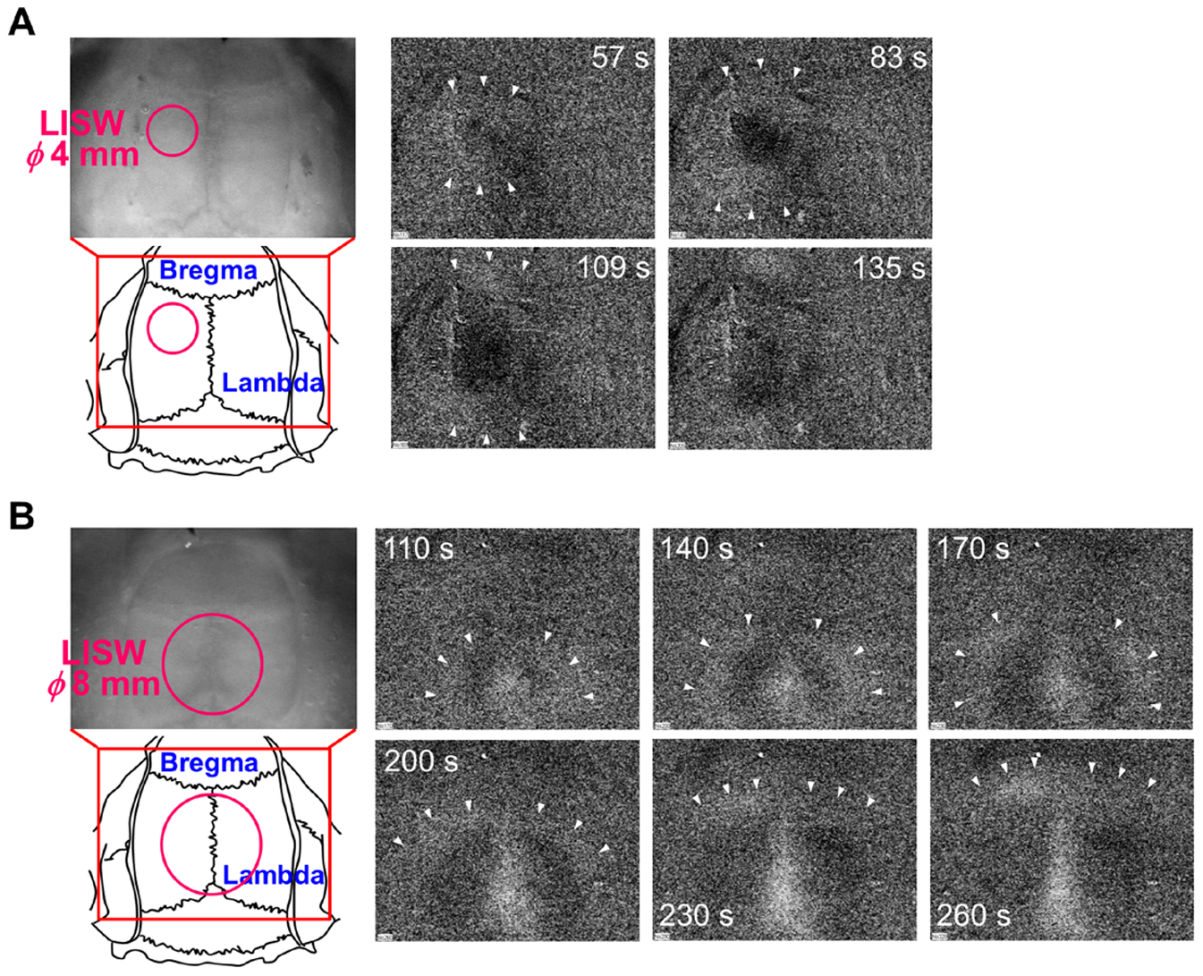
NIR difference reflectance images (video clips) showing propagation of light scattering waves on the rat brain that was exposed to an LISW. Images were acquired with a CCD camera under illumination with a bandpass-filtered tungsten lamp (800±70 nm). The laser target was quickly removed after laser exposure. The LISW was applied at time zero. (A) A 4-mm-diameter LISW generated at 1.0 J/cm^2^ (∼86 MPa; ∼14 Pa•s) was applied to the same location as that for diffuse reflectance measurement (left-most photograph and figure). (B) An 8-mm-diameter LISW generated at 0.7 J/cm^2^ (∼85 MPa; ∼19 Pa•s) was applied to the area around the center of the skull (left-most photograph and figure). In both (A) and (B), arrowheads indicate the front of a bright region(s), which was (were) followed by a dark region(s). Times shown in the NIR difference reflectance images indicate times after LISW application. Propagation speeds in (A) and (B) were 2.4 mm/min and 2.0 mm/min, respectively.

Figure 4A shows the time course of DC potential for the rat brain exposed to an LISW generated at 1.0 J/cm^2^ (∼86 MPa; ∼14 Pa•s). The discontinuous increase at the time of LISW application is attributable to the change of electrode contact with the brain tissue. At ∼2 min after LISW application, the potential started to decrease steeply and continued to decrease for ∼30 s; thereafter it recovered to a certain level in ∼2 min but decreased again and stayed at low levels for a long time. The maximum potential shift was ∼7.2 mV, indicating the occurrence of a strong depolarization. Figure 4B shows the time course of scattering light intensity in the ROI (0.5 mm×1.0 mm) adjacent to the site of the potential measurement, which was simultaneously measured on the basis of CCD imaging. At ∼2.5 min after LISW application, the intensity increased sharply and then dropped; thereafter it gradually recovered to the initial level. The increase and decrease in scattering intensity respectively corresponded to the bright and dark regions in the scattering wave, which are shown later (Fig. 5A). The DC potential shift temporally coincided with the scattering signal change. This scattering change and that obtained by fiber measurement (Fig. 3D) originated from the same phenomenon, although measurement points and observation depths were slightly different. Combining these findings with the EEG suppression shown in Fig. 3E, it can be said that the LISW caused spreading depolarization, which was accompanied by spreading depression and concomitant light scattering change.

DC potential measurement was repeated for five other rats under the same LISW conditions; multiple occurrence of DC potential shift was not observed. Under the higher pressure LISW conditions (1.25 J/cm^2^; ∼104 MPa; ∼19 Pa•s), however, consecutive DC potential shifts were observed in some cases. There might be pressure or impulse-dependent increase in the number of times of occurrence of spreading depolarization/depression (SD).

### Imaging of propagation of scattering wave (SD)

Figure 5A shows difference scattering images (video clips) of the rat brain that was exposed to an LISW under the same conditions as those for the diffuse reflectance measurement (diameter, 4 mm; 1 J/cm^2^; ∼86 MPa; ∼14 Pa•s) (Movie S1). Bright regions appeared at the site of LISW application and they propagated forward and backward over the left hemisphere, and dark regions followed the bright regions. The propagation speed of the scattering waves (SD) was 2.4 mm/min, but they did not get into the contra-hemisphere. When an LISW was applied to the lateral region in the parietal bone or the frontal bone in the same hemisphere, scattering waves were also generated at the site of LISW application and showed similar propagation characteristics (data not shown). When a larger diameter LISW (*φ*8 mm; 0.7 J/cm^2^; ∼85 MPa; ∼19 Pa•s) was applied to the area around the center of the skull, a scattering wave was propagated radially over both hemispheres at ∼2.0 mm/min; the wave covered all of the observed region of the brain. Under this irradiation condition, we occasionally observed that a scattering wave (SD) generated from the occipital lobe, corresponding to the visual cortex, not from the site of LISW application (Figure 5B, Movie S2). There is a similarity between this observation and spreading depression observed in patients with migraine. This issue is discussed later.

### Stereomicroscopic observation of blood vessels in the brain surface

**Figure 6:**
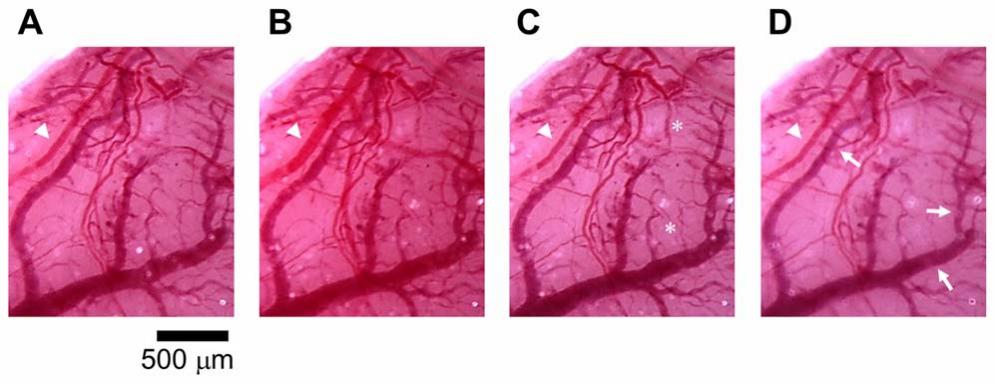
Stereomicroscopic observation of blood vessels in the surface of the brain that was exposed to an LISW. The image was taken through a cranial window of 4-mm-thick PET sheet was fitted. A 4-mm-diameter LISW generated at 1.25 J/cm^2^ (∼104 MPa; ∼19 Pa•s) was applied to the right hand side of the window at time zero. (A) Before LISW application. (B–D) After LISW application: (B) t = 2.5 min, (C) t = 4 min and (D) t = 48 min. Diameters of some pial arteries were smaller than those before LISW application (arrowheads). Tissue color in (C) and (D) was pale. In (D), even some veins showed vasoconstriction (arrows). Some small-diameter vessels seen in (C) (asterisks) were not able to be seen clearly in (D). The state shown in (B) and states shown in (C) and (D) correspond to hyperemia/hyperoxemia and oligemia/hypoxemia, respectively, which were shown by spectroscopic diffuse reflectance measurement (Fig. 3G, F).

Figure 6 shows stereomicroscopic images of blood vessels in the brain surface at four different time points: (A) before LISW application (B), 2.5 min, (C) 4 min and (D) 48 min after LISW application (*φ*4 mm; 1.25 J/cm^2^; ∼104 MPa; ∼19 Pa•s), where the time points (B) and (C, D) correspond to the hyperemic/hyperoximic phase and oligemic/hypoxemic phase, respectively. Pial arteries were remarkably dilated in (B), while they were constricted in (C) and (D); diameters of some arteries were smaller than those before LISW application (arrowheads). In addition, change of tissue color, which reflects diameter of capillaries, was evident. The tissue color in (B) was more reddish than that before LISW application, indicating hyperperfusion in capillaries. On the other hand, the tissue color in (C) and (D) was pale, indicating vasoconstriction of capillaries; vasoconstriction was seen even in some veins (arrows) in (D). Some small-diameter vessels seen in (C) were not able to be seen clearly in (D). These observations indicate that the application of LISW caused a short-lasting vasodilatation, which was followed by a long-lasting vasoconstriction, corresponding respectively to the hyperemia/hyperoxemia and oligemia/hypoxemia observed by the diffuse reflectance measurement (Fig. 3F, G). Thus, hypoxemia observed by the optical measurement would be closely associated with vasoconstriction.

### Spatiotemporal correlation between spreading depression and hypoxemia

**Figure 7:**
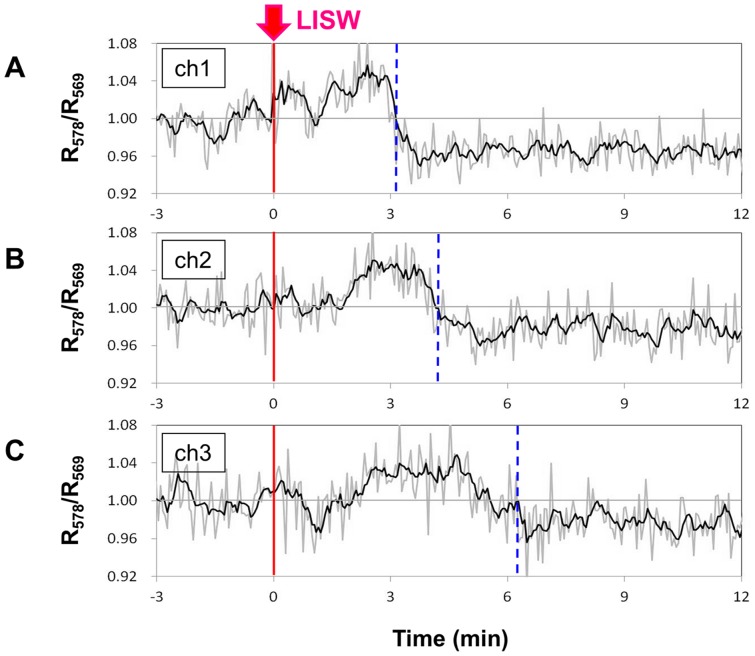
Results of multichannel fiber measurement of hemoglobin oxygenation level. Positions of the fiber pair and LISW application are shown Fig. 2D. An LISW generated at 1.25 J/cm^2^ (*φ*4 mm; ∼104 MPa; ∼19 Pa•s) was applied to the frontal bone at time zero. Time courses of hemoglobin oxygenation (R_578_/R_569_) measured at (A) ch1, (B) ch2 and (C) ch3. The vertical dashed lines indicate turning points from hyperoxemia to hypoxemia.

Figure 7 shows time courses of hemoglobin oxygenation level (R_578_/R_569_) measured at three positions of the fibers: ch1, ch2 and ch3, where time zero indicates the time of LISW application (1.25 J/cm^2^; ∼104 MPa; ∼19 Pa•s). This measurement and the imaging experiment for which results are shown Fig. 6 required different preparations for the rat. Thus, these two experiments were performed separately, although LISW conditions were the same. The hemoglobin oxygenation level at ch1 was very similar to that shown in Fig. 3G; just after LISW application, the oxygenation level increased but began to decrease at ∼3 min and stayed at a low level. At ch2, the oxygenation level started to increase at ∼1.5 min and then started to decrease at ∼4 min. At ch3, time points of increase and decrease in the oxygenation level were further delayed, at ∼2 min and ∼5 min, respectively. These results indicate that region of hypoxemia was expanding over the cortex; its average speed was 2.6 mm/min in this case. We performed the same measurement for three other rats and confirmed reproducibility; the propagation speed of hypoxemia was in the range of 1.9–3.8 mm/min. This coincided with the propagation speed of the scattering wave and hence SD (1.6–3.3 mm/min), showing a strong correlation between SD and hypoxemia-oligemia.

### Quantification of hemoglobin oxygen saturation

**Figure 8:**
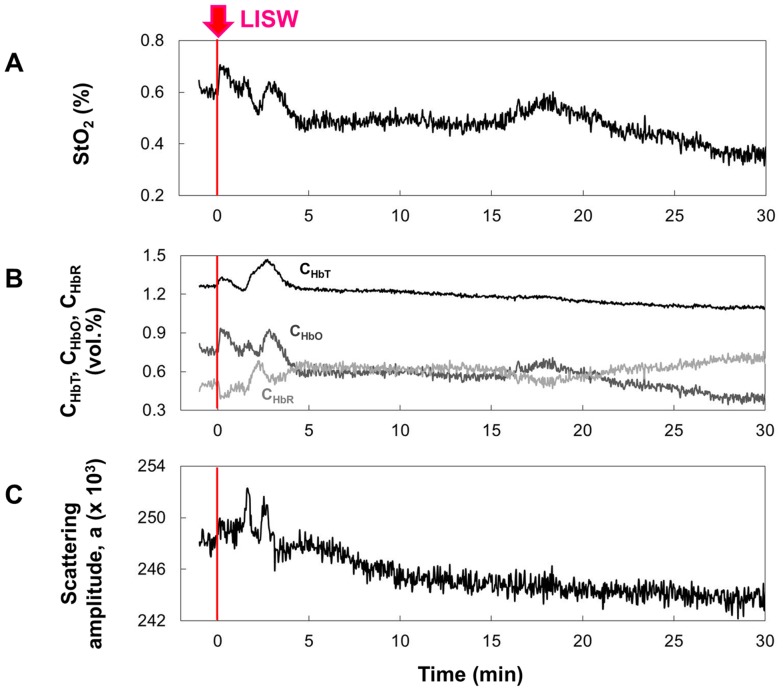
Results of quantification of hemoglobin oxygen saturation (*StO*_2_) based on inverse Monte Carlo simulation. To exclude uncertainty associated with optical properties of the skull, a small window was made in the skull for fiber measurement. Relative location of LISW application (*φ*4 mm; ∼86 MPa; ∼14 Pa•s) and the fiber pair was the same as that for diffuse reflectance measurement (Fig. 2B). Time courses of (A) *StO*_2_ and other important parameters for simulation: (B) concentrations of oxygenated hemoglobin *C*_HbO_, deoxygenated hemoglobin *C*_HbR_ and total hemoglobin *C*_THb_ and (C) scattering amplitude *a*.

Figure 8A shows the results of quantification of hemoglobin oxygen saturation (*StO*_2_) for the rat brain that was exposed to an LISW generated at 1.0 J/cm^2^ (∼86 MPa; ∼14 Pa•s) at time zero (t = 0). For reference, concentrations of oxygenated hemoglobin (*C*_HbO_), deoxygenated hemoglobin (*C*_HbR_) and total hemoglobin (*C*_HbT_) (Fig. 8B) and scattering amplitude (*a*) (Fig. 8C) are also shown. Before LISW application, *StO*_2_ was ∼60%. After LISW application, *StO*_2_ transiently increased to ∼70% but began to decrease at about 4 min after LISW application. There were some fluctuations, but *StO*_2_ continuously showed low values; about 27 min after LISW application, it stayed as low as ∼35%, indicating severe long-lasting hypoxemia. The concentration of oxygenated hemoglobin *C*_HbO_ decreased by ∼50% (Fig. 8B), being consistent with the severe arterial vasoconstriction observed (Fig. 6C, D). Drastic change in the scattering amplitude *a* just after LISW application proved the diffuse reflectance signal change at 805 nm (R_805_).

## Discussion

### Summary and correlation of observations

In this study, we performed real-time measurements of diffuse reflectance signals and EEG for the rat brain that was exposed to a laser-induced shock wave (LISW). Even under conditions in which no clear changes were observed in systemic physiological parameters, we observed a drastic change in the light scattering signal, EEG suppression, long-lasting hypoxemia and signal change indicating mitochondrial energy impairment (Fig. 3). The scattering signal change, which reflected cellular and subcellular morphological changes, and EEG suppression indicated the occurrence of spreading depression, and we examined related events to confirm this. At ∼2 minutes after LISW application, DC potential shifted negatively and this change temporally coincided with the transit of a scattering wave through the site of DC potential measurement (Fig. 4). The scattering wave was observed to propagate over the cortex at a speed of 1.6–3.3 mm/min (Fig. 5). These observations confirmed the occurrence of spreading depolarization. Stereomicroscopic observation of blood vessels in the brain surface showed remarkable vasodilatation for a few minutes after LISW application, which was followed by a long-lasting vasoconstriction, corresponding to the long-lasting hypoxemia (Fig. 6). The region of hypoxemia expanded at a speed similar to that of propagation of the scattering wave (SD) (Fig. 7). Furthermore, computer simulation showed that the hypoxemia observed was severe, ∼40% lower than that before LISW application (Fig. 8).

During spreading depression/depolarization (SD), cells will respond to compensate abnormal influx and efflux of ions through the membranes, and this process consumes much ATP, leading to a loss of tissue viability in the brain. The signal changes indicating mitochondrial energy impairment (Fig. 3H) might be associated with this process. However, the signal changes observed were relatively small, and more sensitive measurement is needed for further discussion, which is an important part of our future study.

### SD and hemodynamic responses

Spreading depolarization/depression (SD) is known to be caused by nonblast TBIs with contusion and hemorrhage. In rodent nonblast TBI models made by fluid percussion (FP) [50]–[53] and controlled cortical impact (CCI) [54]–[57], the occurrence of SD has been shown clearly. Since characteristics of SD depend on many parameters, such as site, size and strength of impact as well as animal conditions, clear-cut description is difficult. However, there seem to be differences in characteristics of SD due to FP injury and those due to CCI injury. In CCI injury, SD is observed immediately after impact with inefficient recurrence and occurs mainly in the ipsilateral (injured) hemisphere. A correlation between SD frequency and intracranial pressure (ICP) is not evident in this model [56]. In FD injury, on the other hand, SD is characterized by delayed and repeated occurrence and is clearly observed even in the contralateral (uninjured) hemisphere [52], [53]; there is a significant correlation between SD frequency and ICP [52]. The mechanism underlying the differences between these two models is not clear, but it seems that SD is directly induced by mechanical impact in CCI injury, while hypoxic or ischemic conditions due to contusion-hemorrhage or increased IPC cause SD in FD injury. Similarity and dissimilarity in SD characteristics and responses between these nonblast TBI models and our shock wave-induced TBI model are discussed later.

In blast-related TBIs, both in animal models [58] and human patients [59], [60], EEG abnormalities have been reported, but it is not clear whether a shock wave itself causes SD in the brain. Under the LISW conditions examined in this study, no or little hemorrhage and contusion were observed in the brain. In addition, changes in systemic physiological parameters were small, indicating limited effects of the LISW on the brainstem or higher controlling center. Even under such mild conditions, we reproducibly observed SD and related long-lasting vasoconstriction and hypoxemia.

Spreading depolarization/depression is known to also occur in various brain diseases, such as stroke, subarachnoid hemorrhage, epilepsy and migraine, and has received much attention as an important signature, as well as a factor determining the severity and prognosis, of the disease [48], [61], [62]. Hemodynamic responses to SD, which can be classified into normal and inverse responses, are important. In the inverse hemodynamic response, long-lasting near-complete depolarization is followed by vasoconstriction in the resistant vessels, leading to severe hypoxia or ischemia (spreading ischemia) [48]. There are common characteristics in our observation for the rat brain exposed to an LISW with those of spreading ischemia. The mechanism of SD-induced vasoconstriction is not fully understood, but a pronounced rise of extracellular potassium ions associated with SD can cause vasoconstriction since potassium at a concentration above ∼20 mM acts as a strong vasoconstrictor [63].

Long-lasting decrease in rCBF has also been reported in rodent nonblast TBI models described above [51]–[53], [55]–[57], but few reports deal with oxygen level and microscopic conditions of blood vessels in the brain. Thus, it is currently difficult to compare the characteristics of SD and its hemodynamic responses in detail between the nonblast TBI models and our shock wave-induced TBI model. As a factor for comparison, the temporal characteristics of depolarization are also important; it has recently been shown that the duration of DC or slow potential negative shift determines pathological outcome in the brain [64]. We can point out some similarities in the spatiotemporal characteristics of SD between CCI injury models and our model, i.e., the immediate occurrence after impact and predominant occurrence in the ipsilateral hemisphere. However, the duration of DC potential shift in our model (Fig. 4) is longer than that reported in the CCI injury model [56], [57], although much more data are needed for reliable comparison.

### Light scattering signal as an indicator of shock wave-induced SD

Our results show that light scattering signals, both by fiber measurement and CCD imaging, are closely associated with SD induced by a shock wave in the brain. The scattering changes are attributable to cellular or subcellular morphological changes, which are caused by efficient influx and efflux of ions though the membrane due to SD. Experiments using brain slice culture showed that cell swelling caused a scattering decrease [65], while organelle swelling or dendritic beading led to a scattering increase [66], [67]. We previously observed that scattering increased in the brain after the anoxic depolarization, and scanning electron microscopy of fixed tissue showed remarkable swelling of mitochondria and dendrites [68]. It should be noted, however, that blood cells are also light scatterers [69], and drastic hemodynamic responses can therefore affect diffuse reflectance from the brain; increased blood flow might cause increased scattering and vice versa. Bright and dark regions observed in the scattering wave (Fig. 5) are attributable to hyperperfusion due to vasodilatation and hypoperfusion due to vasoconstriction (Fig. 6), respectively. However, we observed that vasoconstriction lasted for a long time, while scattering intensity soon recovered to its initial level. Thus, the drastic change in light scattering we observed would reflect both morphological changes of cells and organelles and change of blood flow in the brain. Although further study is needed, our results indicate that the light scattering signal can be an indicator of shock wave-induced SD, which can be monitored noninvasively.

### Systemic exposure versus local exposure

In a real blast injury, the whole body is often exposed to a blast or shock wave, leading not only to diffuse brain injury but also to systemic injury. Systemic injury can considerably affect the brain; in particular, the effect of lung injury on the brain is crucial since it causes systemic hypoxia. It has also been pointed out that impact on the thorax causes vago-vagal reflex. Sudden hyperinflation of the lungs due to thorax impact can stimulate juxta-capillary (J)-receptors located within the alveolar walls, causing vago-vagal reflex. This can lead to apnea followed by rapid breathing, bradycardia and hypotension, thus affecting the brain [70]–[72]. To simulate systemic blast injury, whole body exposure is needed, for which large-bore shock/blast tubes are useful. However, events caused by such whole body exposure are extremely complex, and an inductive approach would therefore also be useful to investigate the pathophysiology and mechanism of bTBIs. From this viewpoint, an LISW would be a useful tool, by which pure effects of a shock wave on the brain can be examined under various conditions. Thoracic mechanisms, including that described above, would be associated with mild bTBI (mbTBI), but the results of our study show that application of a shock wave to the brain solely causes SD and related long-lasting vasoconstriction and hypoxemia in the brain. This inverse hemodynamic response-like phenomenon can cause neuronal cell death, suggesting that mbTBI is also caused by direct cranial transmission of a shock wave, without thoracic impact.

### Factors affecting experimental outcome and clinical implications

In animal experiments relating to TBI, type of anesthesia used possibly affects the outcome because of its neuroprotective effect [73]. In the experiments described above, we used anesthesia with pentobarbital sodium. To check the effect of the type of anesthesia, we also performed the same diffuse reflectance measurement for rats under anesthesia by isoflurane inhalation (5% isoflurane in air); there is a report on a more potent neuroprotective effect than that of pentobarbital sodium [73]. We observed similar SD in this experiment; type of anesthesia seems not to greatly affect the occurrence of SD itself, although downstream events, such as neuronal cell death, may be changed. In real blast injuries, a neuroprotective effect due to anesthesia is, of course, not expected, and the outcome might thus be different.

Type of animals can also affect the outcome. It is known that SD occurs more easily in lower species [74]; for humans, threshold energy of a shock wave to induce SD would be higher. However, many common outcomes and complications of SD in animals and humans have been reported [75], [76]. From a mechanical point of view, the human skull is much thicker (3∼8 mm) than the rat skull (∼1 mm), and human brain is therefore much more efficiently protected against a shock wave. However, the human skull, even with a helmet, cannot perfectly reflect a coming shock wave; a part of the shock wave energy must penetrate through the skull to reach the brain. The face, e.g., the forehead, can be a transcranial window for a shock wave to reach the brain when a helmet is worn. The influence simply depends on the pressure amplitude of the shock wave. When the coming pressure amplitude is high enough, a shock wave that has penetrated through the skull can considerably affect the brain.

In our experiments using rats, SD was generated at the site of LISW application with a diameter of 4 mm and then propagated over the cortex in the hemisphere, indicating that the focal effect of a shock wave can affect a much wider region in the brain. When a larger diameter (8 mm) LISW was applied to the center of the parietal bone, however, SD was occasionally generated in the visual cortex (Fig. 5B). There is a similarity in this observation to observations of SD in patients with migraine [77]. Spreading depolarization/depression is caused and accelerated by efflux of potassium ions or glutamate, while glial cells can buffer them [78]–[80]. Thus, the density of glial cells in tissue is associated with the ease of occurrence of SD. Since it is known that the glial cell to neuron ratio is relatively low in the visual cortex [81], the threshold of SD occurrence in this region becomes lower than those in other regions in the brain. There seems to be a similar mechanism in our observation described above. Recently, Goodrich et al. compared vision problems in nonblast and blast-related TBI patients and reported that light sensitivity (photophobia) was observed in a significantly larger proportion of bTBI patients (67%) than nonblast TBI patients (33%) (p<0.01) [82]. The reason for this is unknown, but the probability of SD occurrence, particularly in the visual cortex, might be different between the two groups, resulting in the different outcomes of vision problems.

In recent years, clinical studies have been conducted to investigate the characteristics and outcomes of SDs that are induced by brain injuries, including nonblast TBIs [64], [83]–[85]. Fabricious et al. reported the occurrence of SD in 4 out of 5 TBI patients [83]. Hartings et al. studied 53 acute TBI patients and reported that 30 (57%) of the patients exhibited spreading depolarization and their outcomes critically depended on the characteristics of spreading depolarization, such as durations of DC shift and high-frequency electrocorticographic depression [64]. It should be noted, however, that TBIs in all of these patients were severe, for which neurosurgery was performed. For the patients, an electrode was placed on the brain during or after surgery and then measurement was started; there were no data during at least a few tens of hours after injury. Although mean arterial pressure, cerebral perfusion pressure and ICP were recorded for the patients, data on oxygen level and microscopic vasculature conditions were not reported. Continuous efforts will soon reveal more details of the hemodynamic responses of SD and their outcomes in TBI patients.

Despite the strong interest in SD for various brain diseases including nonblast TBIs as described above, SD and related events have not been discussed in detail in reports on bTBI. Vasoconstriction or vasospasm has been recognized as a common complication in patients with moderate or severe bTBI [5], [9], [86], but its mechanism is unclear. The results of this study indicate the possibility that shock wave-induced SD is associated with this common complication.

### LISW-based rat bTBI models

Shock wave-induced SD or related vasoconstriction can be an important therapeutic target for bTBI. To investigate the possible therapeutic strategy, an animal model is needed. To the authors' knowledge, however, no animal model has been established for shock wave-induced SD and related hemodynamic responses. Although there are various animal models for SD, e.g., electrical stimulation [87], [88], KCl application/injection [88]–[91], pinprick [92]–[94], hypoxia [75], [95] and ischemia [88], [89], [96], their characteristics and outcomes should be different. Our rats with application of an LISW to the brain can be used as a reliable animal model to reproduce SD and related events. In parallel with the present real-time monitoring experiment, we are conducting pathological examination and behavioral analysis for the rats. We observed axonal injury, as well as behavioral impairment (depression and anxiety), under certain conditions; the results will be reported elsewhere. Understanding the correlation between the results of these experiments will provide a new insight into the mechanism of bTBI.

## Supporting Information

Movie S1 **Movie showing propagation of light scattering waves on the rat brain that was exposed to an LISW.** A 4-mm-diameter LISW generated at 1.0 J/cm2 (∼86 MPa; ∼14 Pa•s) was applied to the same location as that for diffuse reflectance measurement (left-most photograph and figure in Fig. 5A).
(AVI)

Movie S2 **Movie showing propagation of light scattering waves on the rat brain that was exposed to an LISW.** An 8-mm-diameter LISW generated at 0.7 J/cm2 (∼85 MPa; ∼19 Pa•s) was applied to the area around the center of the skull (left-most photograph and figure in Fig. 5B).
(AVI)
