# Correction: Real-Time Optical Diagnosis of the Rat Brain Exposed to a Laser-Induced Shock Wave: Observation of Spreading Depolarization, Vasoconstriction and Hypoxemia-Oligemia

**DOI:** 10.1371/journal.pone.0095067

**Published:** 2014-04-09

**Authors:** The PLOS ONE Staff

The term “R_578_/R_569_” appears incorrectly in several locations throughout the manuscript. The following instances of “R_578_/R_569_” should appear as “1+(1- R_578_/R_569_)”:

The fourth sentence of the second paragraph of the “Real-time optical diagnosis of the rat brain” section of the Materials and Methods, the second paragraph of the “Systemic physiology, EEG and diffuse reflectance signals for the brain” section of the Results, and the vertical axis and legend for Figure 3G. The correct version of Figure 3 is below.

**Figure 3:**
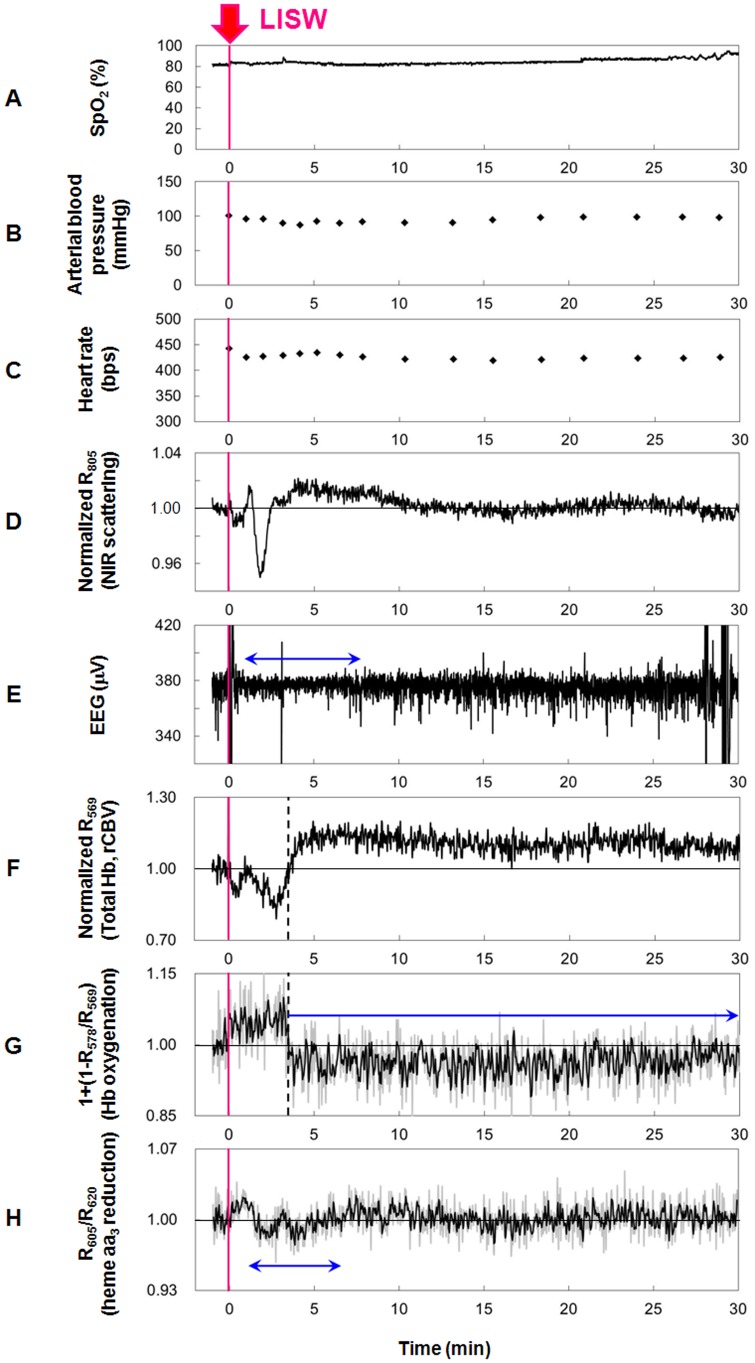
Results of measurements of systemic physiological parameters, EEG and diffuse reflectance signals for the brain. Sensor positions for systemic physiological parameters (A–C), EEG (E) and diffuse reflectance signals (D, F–H) are shown in Fig. 2. A single pulse of LISW generated at 1.0 J/cm^2^ (*φ*4 mm; ∼86 MPa; ∼14 Pa
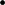
s) was applied to the brain at time zero. (A) Arterial oxygen saturation (SpO_2_). (B) Arterial blood pressure. (C) Heart rate. (D) Light scattering signal (diffuse reflectance signal at 805 nm, R_805_) indicating cellular and subcellular morphological changes. (E) EEG. The horizontal arrow indicates the duration of EEG suppression. (F) Total hemoglobin indicating regional cerebral blood volume (rCBV) (R_569_). The vertical dashed line indicates the turning point from hyperemia to oligemia. (G) Hemoglobin oxygenation (1+(1-R_578_/R_569_)). The vertical dashed line indicates the turning point from hyperoxemia to hypoxemia. The horizontal arrow indicates long-lasting hypoxemia. (H) Diffuse reflectance signal indicating reduction of heme aa_3_, a redox center of cytochrome c oxidase (R_605_/R_620_). The horizontal arrow indicates the duration of heme aa_3_ reduction.

The term “R_620_/R_605_” appears incorrectly in the legend for Figure 3. The correct term is “R_605_/R_620_.” The correct legend for Figure 3 is below.

The following occurrences of the term “R_578_/R_569_” are incorrect and should appear as “R_560_/R_569_”:

The last sentence of the “Spatiotemporal correlation between spreading depression and hypoxemia” section of the Materials and Methods, the “Spatiotemporal correlation between spreading depression and hypoxemia” section of the Results, and the vertical axis and legend for Figure 7. The correct version of Figure 7 is below.

**Figure 7:**
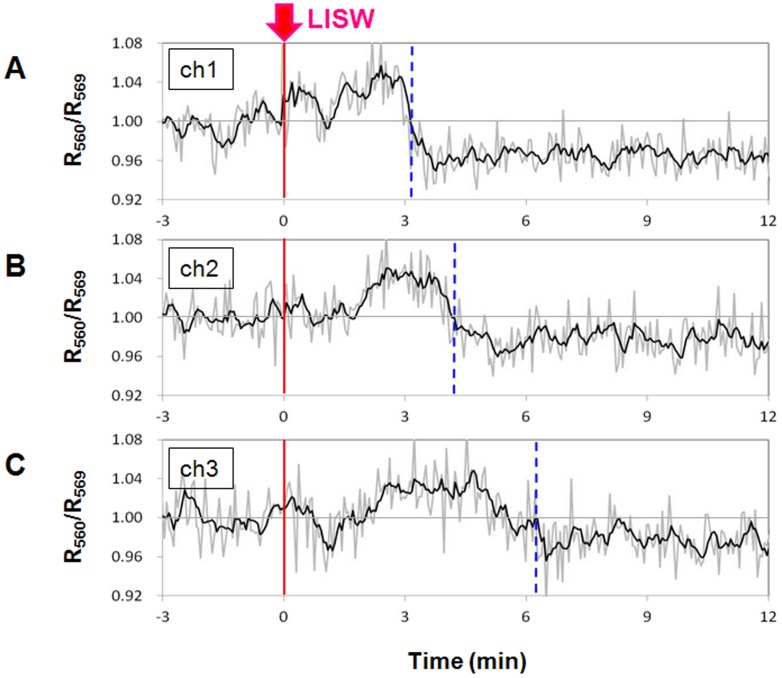
Results of multichannel fiber measurement of hemoglobin oxygenation level. Positions of the fiber pair and LISW application are shown Fig. 2D. An LISW generated at 1.25 J/cm^2^ (*φ*4 mm; ∼104 MPa; ∼19 Pa
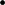
s) was applied to the frontal bone at time zero. Time courses of hemoglobin oxygenation (R_560_/R_569_) measured at (A) ch1, (B) ch2 and (C) ch3. The vertical dashed lines indicate turning points from hyperoxemia to hypoxemia.

The term “C_THb_” appears incorrectly in the legend for Figure 8. The correct term is “C_HbT_”.
